# Risk of further decompensation/mortality in patients with cirrhosis and ascites as the first single decompensation event

**DOI:** 10.1016/j.jhepr.2022.100513

**Published:** 2022-06-03

**Authors:** Lorenz Balcar, Marta Tonon, Georg Semmler, Valeria Calvino, Lukas Hartl, Simone Incicco, Mathias Jachs, David Bauer, Benedikt Silvester Hofer, Carmine Gabriele Gambino, Antonio Accetta, Alessandra Brocca, Michael Trauner, Mattias Mandorfer, Salvatore Piano, Thomas Reiberger, Baveno Cooperation: an EASL consortium

**Affiliations:** 1 Division of Gastroenterology and Hepatology, Department of Internal Medicine III, Medical University of Vienna, Vienna, Austria; 2 Vienna Hepatic Hemodynamic Lab, Division of Gastroenterology and Hepatology, Department of Internal Medicine III, Medical University of Vienna, Vienna, Austria; 3 Unit of Internal Medicine and Hepatology, Department of Medicine, University of Padova, Padua, Italy; 4 Christian-Doppler Laboratory for Portal Hypertension and Liver Fibrosis, Medical University of Vienna, Vienna, Austria

**Keywords:** Portal hypertension, Hepatic decompensation, Spontaneous bacterial peritonitis, Acute kidney injury, Baveno, ACLF, acute-on-chronic liver failure, aSHR, adjusted subdistribution hazard ratio, CI, confidence interval, CPS, Child–Pugh score, HCC, hepatocellular carcinoma, HE, hepatic encephalopathy, HRS-AKI, hepatorenal syndrome–acute kidney injury, ICA, International Club of Ascites, INR, international normalised ratio, IQR, interquartile range, LT, liver transplantation, MELD, model for end-stage liver disease, NAFLD, non-alcoholic fatty liver disease, NASH, non-alcoholic steatohepatitis, PVT, portal vein thrombosis, RA, refractory ascites, SBP, spontaneous bacterial peritonitis, SHR, subdistribution hazard ratio, TFS, transplant-free survival, TIPS, transjugular intrahepatic portosystemic shunt, UNOS MELD (2016), United Network for Organ Sharing model for end-stage liver disease (2016), VB, variceal bleeding

## Abstract

**Background & Aims:**

Although ascites is the most frequent first decompensating event in cirrhosis, the clinical course after ascites as the *single* index decompensation is not well defined. The aim of this multicentre study was thus to systematically investigate the incidence and type of further decompensation after ascites as the first decompensating event and to assess risk factors for mortality.

**Methods:**

A total of 622 patients with cirrhosis presenting with grade 2/3 ascites as the *single* index decompensating event at 2 university hospitals (Padova and Vienna) between 2003 and 2021 were included. Events of further decompensation, liver transplantation, and death were recorded.

**Results:**

The mean age was 57 ± 11 years, and most patients were male (n = 423, 68%) with alcohol-related (n = 366, 59%) and viral (n = 200,32%) liver disease as the main aetiologies. In total, 323 (52%) patients presented with grade 2 and 299 (48%) with grade 3 ascites. The median Child–Pugh score at presentation was 8 (IQR 7–9), and the mean model for end-stage liver disease (MELD) was 15 ± 6. During a median follow-up period of 49 months, 350 (56%) patients experienced further decompensation: refractory ascites (n = 130, 21%), hepatic encephalopathy (n = 112, 18%), spontaneous bacterial peritonitis (n = 32, 5%), hepatorenal syndrome–acute kidney injury (n = 29, 5%). Variceal bleeding as an isolated further decompensation event was rare (n = 18, 3%), whereas non-bleeding further decompensation (n = 161, 26%) and ≥2 concomitant further decompensation events (n = 171, 27%) were frequent. Transjugular intrahepatic portosystemic shunt was used in only 81 (13%) patients. In patients presenting with grade 2 ascites, MELD ≥15 indicated a considerable risk for further decompensation (subdistribution hazard ratio [SHR] 2.18; *p* <0.001; 1-year incidences: <10: 10% *vs*. 10–14: 13% *vs*. ≥15: 28%) and of mortality (SHR 1.89; *p* = 0.004; 1-year incidences: <10: 3% *vs*. 10–14: 6% vs. ≥15: 14%). Importantly, mortality was similarly high throughout MELD strata in grade 3 ascites (*p* = n.s. for different MELD strata; 1-year incidences: <10: 14% *vs.* 10–14: 15% *vs*. ≥15: 20%).

**Conclusions:**

Further decompensation is frequent in patients with ascites as a *single* index decompensation event and only rarely owing to bleeding. Although patients with grade 2 ascites and MELD <15 seem to have a favourable prognosis, those with grade 3 ascites are at a high risk of mortality across all MELD strata.

**Lay summary:**

Decompensation (the development of symptoms as a result of worsening liver function) marks a turning point in the disease course for patients with cirrhosis. Ascites (*i.e.,* the accumulation of fluid in the abdomen) is the most common first decompensating event, yet little is known about the clinical course of patients who develop ascites as a *single* first decompensating event. Herein, we show that the severity of ascites is associated with mortality and that in patients with moderate ascites, the widely used prognostic MELD score can predict patient outcomes.

## Highlights

• A course of patients with first *single* ascites decompensation has so far not been described.
• MELD score is important for risk prediction/stratification in patients with grade 2 ascites but not grade 3 ascites.
• A threshold of MELD at 15 points discriminates patients with favourable/poor outcome in patients with grade 2 ascites.
• In patients with grade 3 ascites, regardless of MELD, treatment strategies should be intensified.

## Graphical abstract

**Figure:**
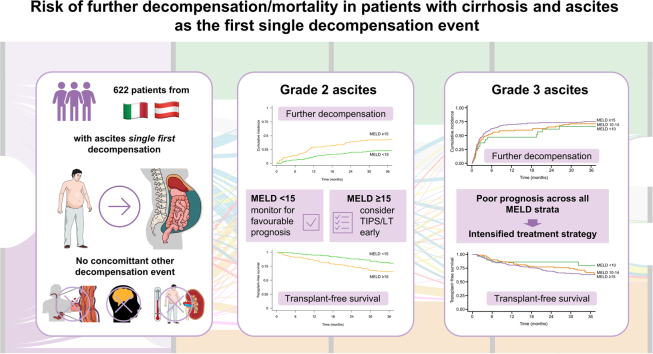

## Introduction

Ascites is the most common decompensating event with an anual incidence rate of 5-10% in patients with cirrhosis.[1], [2], [3] While ascites does not only lead to a reduction in quality of life,^4^^,^^5^ it is also associated with considerable morbidity and mortality.^1^ After development of ascites, further decompensating events may occur that can be sub-classified as being ascites-related (*i.e.,* spontaneous bacterial peritonitis [SBP], dilutional hyponatraemia, and hepatorenal syndrome–acute kidney injury [HRS-AKI][6], [7], [8]) or non-ascites-related (*i.e.,* variceal bleeding and hepatic encephalopathy [HE]) may follow and subsequently impact on the clinical course.^3^

According to the Baveno VII consensus, the treatment paradigm of ascites changed from controlling (*i.e.,* treating) ascites to the prevention of further decompensation (*i.e.,* treating the underlying mechanisms of disease progression) and mortality.^9^ This might be achieved by aetiological treatment,[10], [11], [12], [13], [14] long-term albumin administration,^15^ transjugular intrahepatic portosystemic shunt (TIPS) implantation,^16^ or liver transplantation (LT). To this end, comprehensive and representative data on the clinical course after ascites development are needed but currently very limited.^1^^,^^2^ Therefore, the aim of this multicentre study was to systematically investigate the incidence and type of further decompensation after ascites as the first *single* decompensating event and to assess risk factors for mortality.

## Patients and methods

### Study design and population

Patients with cirrhosis and ascites as the first and only index decompensating event followed up at the University Hospital of Padova (Italy) and the General Hospital of Vienna/Medical University of Vienna (Austria) between 2003 and Q1/2021 were considered eligible for this retrospective study. Overall, 3,377 patients with cirrhosis were considered for inclusion (n = 1,049 patients from Padova and n = 2,328 patients from Vienna). Patients were excluded if they had grade 1 ascites or any of the following criteria were present: active intrahepatic or extrahepatic malignancy, any previous decompensation (portal hypertensive bleeding and HE), significant cardiovascular or pulmonary disease, chronic kidney disease requiring renal replacement therapy, AIDS, history of LT, occlusive portal vein thrombosis (PVT), patients with liver failure at baseline (defined as bilirubin ≥12 mg/dl), patients with rare/non-cirrhotic aetiologies of ascites (*i.e.,* Wilson disease, alpha-1 antitrypsin deficiency-related liver disease, Budd–Chiari syndrome, porto-sinusoidal vascular disease, or other vascular liver diseases), or insufficient clinical data.

### Recorded parameters

Ascites was graded as moderate (grade 2) or severe (grade 3) according to the International Club of Ascites (ICA) guidelines.^17^ Patients with mild ascites (*i.e.,* grade 1) were not considered. Demographic, clinical, and relevant laboratory parameters were collected and the Child–Pugh score (CPS) as well as United Network for Organ Sharing (UNOS) model for end-stage liver disease (MELD) (2016) were calculated. The UNOS MELD (2016) score was used throughout this paper, so the MELD score always refers to the UNOS MELD (2016) score. The following endpoints were evaluated during follow-up: development of (i) refractory ascites according to ICA criteria,^17^ (ii) SBP, (iii) HRS-AKI, (iv) HE, (v) variceal bleeding, (vi) hyponatraemia <130 mmol/L, (vii) acute-on-chronic liver failure (ACLF), (viii) hepatocellular carcinoma (HCC), (ix) TIPS implantation, (x) LT, and (xi) PVT. Further decompensation was defined according to Baveno VII criteria^9^ as the development of refractory ascites, SBP, HRS-AKI, HE, or variceal bleeding during follow-up.

### Statistical analysis

Statistical analyses were performed using R 4.1.2 (R Core Team, R Foundation for Statistical Computing, Vienna, Austria). Categorical variables were reported as absolute (n) and relative frequencies (%), whereas continuous variables as mean ± SD or median (IQR), as appropriate. Student’s *t* test was used for group comparisons of normally distributed variables and the Mann–Whitney *U* test for non-normally distributed variables. Group comparisons of categorical variables were performed using either Pearson’s Chi-square or Fisher’s exact test. The median follow-up time was calculated using the reverse Kaplan–Meier method from the date of inclusion to the LT/death/last follow-up date. Incidence of further hepatic decompensation/death was shown as cumulative incidence according to a Fine–Gray competing risk regression model considering LT (± death) as competing risk(s) and considering both centres as a clustering variable.^18^ Next, variables that were significantly different among baseline characteristics, significantly associated with the outcome of interest in univariable analysis, or we considered highly relevant for the prediction of the endpoint of interest were included into a multivariable competing risk model as covariables. A Sankey plot was used for graphical representation of the different courses of disease during follow-up.

A *p* value of <0.05 was considered statistically significant.

### Ethics

The study was conducted in accordance with the principles of the Declaration of Helsinki and was approved by the local ethic committees (EK1008/2011, EK1262/2017, and 0013337/2022). The requirement of written informed consent for the retrospective study cohort was waived by the ethics committees.

## Results

### Patient characteristics at index decompensation

After the inclusion and exclusion criteria were applied, 622 patients were included in our study (n = 315 from Padova and n = 307 patients from Vienna; Fig. S1).

The mean age was 56.5 ± 11.2 years, and most patients were male (n = 423, 68%; Table 1).

**Table 1:** **Patient characteristics at index decompensation in the overall cohort (n = 622)**.

	Value
Patients, n (%)	622 (100%)
Age (years), mean ± SD	56.5 ± 11.2
Sex (male), n (%)	423 (68%)
Aetiology∗, n (%)	
Alcohol-related liver disease	366 (59%)
HCV	142 (23%)
HBV	58 (9%)
NAFLD	75 (12%)
Autoimmune	30 (5%)
Other	36 (6%)
Ascites grade, n (%)	
Grade 2/moderate	323 (52%)
Grade 3/severe	299 (48%)
Varices† (yes *vs.* no), n (%)	
None	193 (31%)
Small	198 (32%)
Large	204 (33%)
Child–Pugh stage, n (%)	
A	93 (15%)
B	359 (58%)
C	170 (27%)
Child–Pugh score, median (IQR)	8 (7–9)
MELD, mean ± SD	15.1 ± 5.9
Non-selective beta-blocker, n (%)	224 (36%)
Diuretics, n (%)	554 (89%)
Furosemide	53 (9%)
Anti-aldosteronic	125 (20%)
Both	376 (61%)
Albumin (g/L), mean ± SD	32.2 ± 6.1
Bilirubin (mg/dl), median (IQR)	1.96 (1.10–3.55)
INR, mean ± SD	1.42 ± 0.32
Platelets (g/L), median (IQR)	114 (72–164)

INR, international normalised ratio; MELD, model for end-stage liver disease; NAFLD, non-alcoholic fatty liver disease.

∗ Patients may have more than 1 liver disease aetiology.

† Missing information in 27 (4%) patients.

At baseline, 323 patients (52%) presented with grade 2 ascites and 299 (48%) with grade 3 ascites. Varices were present in 68% of patients (n = 402; n = 198 [33%] small and n = 204 [34%] large varices). The median CPS was 8 (IQR 7–9). Most patients (58%, n = 359) were classified as CPS-B and 27% (n = 170) as CPS-C at index ascites decompensation, but there were also some patients classified as CPS-A (15%; n = 93). The mean MELD was 15.1 ± 5.9 with 122 patients (20%) presenting with MELD <10, 183 patients (30%) with MELD 10–14, and 306 patients (50%) with MELD ≥15 points. Overall, 224 patients (36%) received non-selective beta-blocker medication at study inclusion, specifically 37% patients (n = 121) with grade 2 ascites and 31% patients (n = 105) with grade 3 ascites. In addition, 89% of patients (n = 554) received diuretics at study inclusion: 86% (n = 277) of patients with grade 2 ascites and 93% (n = 277) with grade 3 ascites. In total, 9% (n = 53) had furosemide and 20% (n = 125) aldosterone antagonists as monotherapy, whereas 61% of patients (n = 376) received the combination of both medications. The median platelet count was 114 (IQR 72–164) G/L, mean albumin levels were 32.2 ± 6.1 g/L, and median bilirubin values were 1.96 (IQR 1.10–3.55) mg/dl.

### Clinical course after index decompensation

**Fig. 1:**
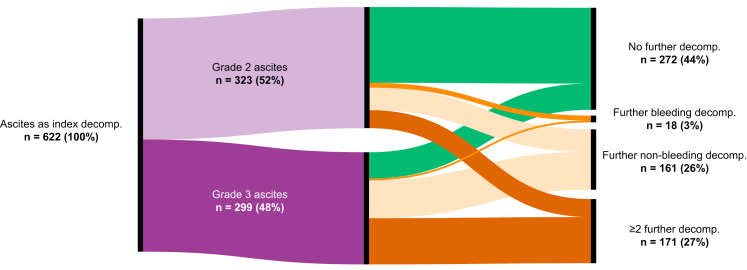
Clinical course after ascites as the index decompensation event. A Sankey plot of the first further decompensation event in patients with ascites as the first index decompensation.

**Fig. 2:**
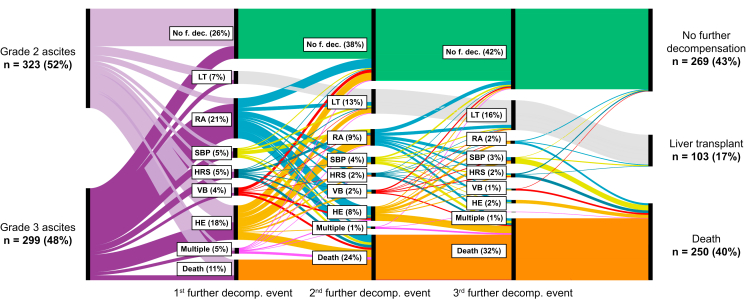
Temporal sequence of further decompensating events. A Sankey plot depicting the temporal sequence of further decompensating events according to ascites graduation and outcome at the last follow-up. HRS, hepatorenal syndrome (-AKI, acute kidney injury); LT, liver transplantation; multiple, more than 1 concomitant decompensating event; no f. dec., no further decompensation events; RA, refractory ascites; SBP, spontaneous bacterial peritonitis; VB, variceal bleeding.

During follow-up, 350 patients (56%) developed any further decompensation after a median of 11.3 (IQR 2.5–36.0) months (Table 2). Among the 323 patients with grade 2 ascites, 149 patients (46%) and 39 patients (12%) progressed to grade 3 and refractory ascites. In addition, 55% (n = 165) of patients with grade 3 ascites progressed to refractory ascites. In general, patients with grade 3 ascites had a median of 4 (1–9) paracenteses during follow-up. In patients with grade 3 ascites who did not experience further decompensation, the median number of paracenteses was only 1 (0–1) *vs.* 5 (3–12) paracenteses in patients with grade 3 ascites who did experience further decompensation. The types of the first further decompensation event in patients who developed further decompensation were refractory ascites (n = 130, 21%), HE (n = 112, 18%), SBP (n = 32, 5%), HRS-AKI (n = 29, 5%), and variceal bleeding (n = 27, 4%). When looking at individual decompensation events, refractory ascites occurred in 204 (33%, 1-/3-year incidences: 24%/30%), HE in 183 (29%, 1-/3-year incidences: 19%/28%), SBP in 105 (17%, 1-/3-year incidences: 10%/15%), HRS-AKI in 81 (13%, 1-/3-year incidences: 8%/12%), and variceal bleeding in 54 patients (9%, 1-/3-year incidences: 4%/8%). Cumulative incidences of each further decompensating event are shown in Fig. S2. Importantly, variceal bleeding as an isolated further decompensation without any other further decompensation event was rare (n = 18, 3%), whereas further non-bleeding decompensation (n = 161, 26%) and ≥2 further decompensation events during follow-up (n = 171, 27%) were common (Fig. 1). Fig. 2 depicts a temporal sequence of further decompensation events graphically using a Sankey plot.

**Table 2:** **Clinical course after ascites as index decompensation event in the study cohort (n = 622)**.

	Value
Patients, n (%)	622 (100%)
Time of follow-up (months), median (IQR)	71.5 (62.0–80.5)
Any *further* decompensation, n (%)	350 (56%)
First *further* decompensation event, n (%)	
Refractory ascites	130 (21%)
SBP	32 (5%)
HRS-AKI	29 (5%)
Variceal bleeding	27 (4%)
Hepatic encephalopathy	112 (18%)
More than one decompensation event	20 (3%)
All *further* decompensation events, n (%)	
Refractory ascites	204 (33%)
SBP	105 (17%)
HRS-AKI	81 (13%)
Variceal bleeding	54 (9%)
Hepatic encephalopathy	183 (29%)
Hyponatraemia∗, n (%)	176 (28%)
ACLF, n (%)	146 (23%)
PVT, n (%)	62 (10%)
HCC, n (%)	79 (13%)
TIPS, n (%)	81 (13%)
LT, n (%)	105 (17%)
Death, n (%)	262 (42%)
Liver-related	224 (85%)
Non-liver-related	36 (14%)
Unknown	2 (1%)

ACLF, acute-on-chronic liver failure; HCC, hepatocellular carcinoma; HRS-AKI, hepatorenal syndrome–acute kidney injury; LT, liver transplantation; PVT, portal vein thrombosis; SBP, spontaneous bacterial peritonitis; TIPS transjugular intrahepatic portosystemic shunt.

∗ Defined as serum sodium <130 mmol/L.

Hyponatraemia, as defined by sodium values <130 mmol/L, was observed in 46 patients (7%) at baseline and 176 patients (28%) during follow-up when excluding patients with hyponatraemia at baseline. HCC occurred in 79 patients (13%). Almost every 4th patient developed ACLF during follow-up (n = 146, 23%), whereas every 10th patient developed PVT (n = 62, 10%). After a median time of 21.4 (IQR 8.0–52.0) and 25.1 (IQR 10.5–59.1) months, TIPS was placed in 81 patients (13%) and 97 patients (16%) underwent LT, respectively.

In total, 262 patients (42%) died. Of these, 224 deaths (85%) were liver-related, whereas only 36 (14%) were not. Cumulative incidence plots for death and LT are presented in Fig. S3.

### Comparison of the clinical course between grade 2 and grade 3 ascites

When comparing the clinical course of patients with grade 2 ascites with that of patients with grade 3 ascites, we found that development of further decompensation was more common in patients with grade 3 ascites (77% *vs*. 37% in grade 2 ascites; subdistribution hazard ratio [SHR] 3.64; 95% CI 2.93–4.51; *p* <0.001; Table S1). Patients with grade 3 ascites had worse prognostic scores than patients with grade 2 ascites (CPS 7 [IQR 6–9] *vs*. 9 [IQR 8–10] points; *p* <0.001; MELD 13.6 ± 5.4 *vs*. 16.8 ± 6.0 points; *p* <0.001). In detail, the cumulative incidences of individual further decompensation events (only considering the first event that occurred) and any further decompensation event were considerably higher in patients with grade 3 than in those with grade 2 ascites, as provided in Table S1. The risks of refractory ascites (SHR 6.90; 95% CI 4.92–9.67; *p* <0.001), SBP (SHR 3.36; 95% CI 2.22–5.08; *p* <0.001), HRS-AKI (SHR 2.96; 95% CI 1.86–4.72; *p* <0.001), and HE (SHR 2.08; 95% CI 1.56–2.78; *p* <0.001) were higher in grade 3 ascites than in grade 2 ascites, whereas the risks of variceal bleeding development (SHR 1.42; 95% CI 0.82–2.45; *p* = 0.210) and PVT (SHR 1.01; 95% CI 0.62–1.65; *p* = 0.980) were not significantly different. In line, hyponatraemia (SHR 2.08; 95% CI 1.59–2.71; *p* <0.001) and ACLF (SHR 3.08; 95% CI 2.18–4.36; *p* <0.001) were more common in grade 3 ascites. As shown in Fig. S4A, the cumulative incidence of further decompensation was significantly different when stratifying patients according to ascites grade (3 *vs*. 2; SHR 3.64; 95% CI 2.93–4.51; *p* <0.001). This could also be demonstrated for transplant-free survival (TFS; 3 *vs*. 2; SHR 1.40; 95% CI 1.10–1.79; *p* = 0.006) depicted in Fig. S4B.

### Further decompensation and TFS

**Fig. 3:**
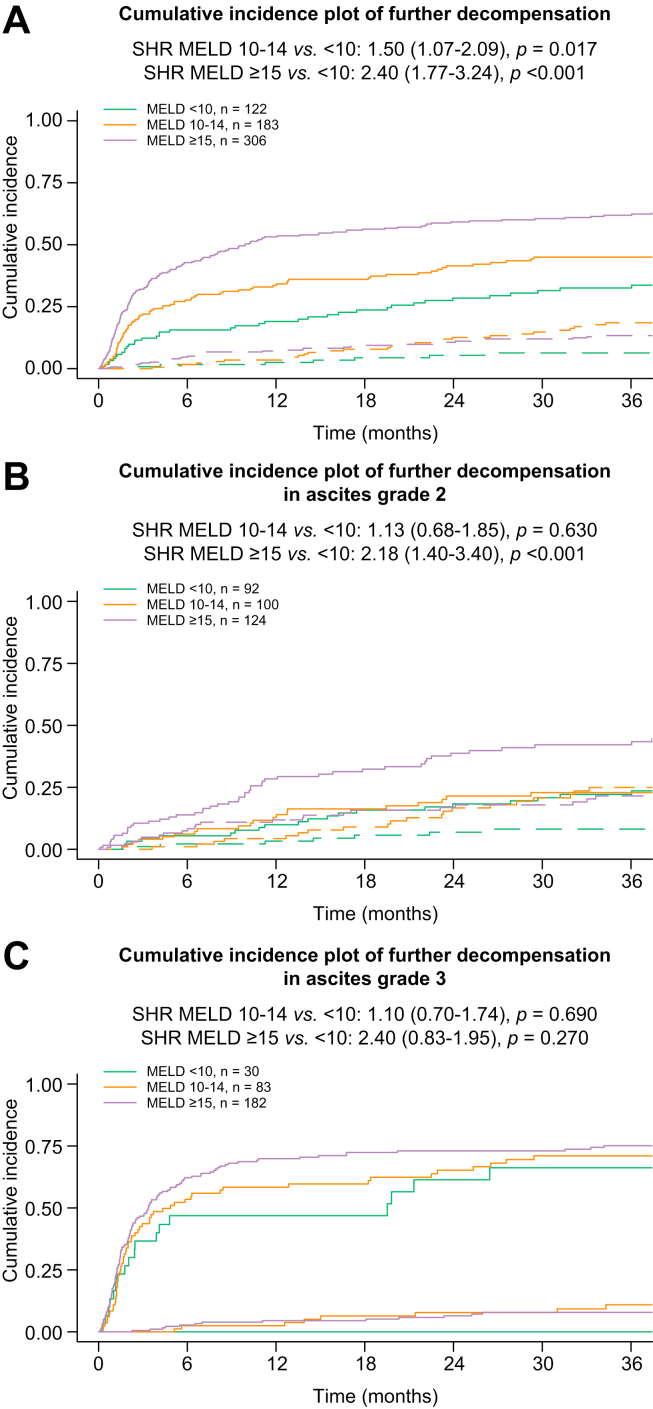
Further decompensation according to MELD and ascites severity at index presentation. Cumulative incidence plot of further decompensation stratified according to MELD in (A) the study cohort, (B) patients with grade 2 ascites, and (C) patients with grade 3 ascites. MELD, model for end-stage liver disease; SHR, sub-distribution hazard ratio.

Stratifying patients according to MELD revealed patient groups with distinct risks of further decompensation after 3 years: <10: 33% *vs.* 10–14: 45% *vs.* ≥15 points: 62% (*p* <0.001). In competing risk analysis, the comparison of MELD 10–14 with <10 points (SHR 1.50; 95% CI 1.07–2.09; *p* = 0.017) as well as ≥15 with <10 points (SHR 2.40; 95% CI 1.77–3.24; *p* <0.001) revealed significant differences in the development of further decompensation (Fig. 3A). In patients with grade 2 ascites, MELD <15 points identified patients with intermediate risks (<10: 28%; 10–14: 32%; SHR: 1.13; 95% CI 0.68–1.85; *p* = 0.630), whereas MELD ≥15 identified patients with high risks of further decompensation after 3 years (42%; ≥15 *vs.* <10: SHR 2.18; 95% CI 1.40–3.40; *p* <0.001; Fig. 3B). Interestingly, in patients with grade 3 ascites at index decompensation, the incidence of further decompensation was similarly high, regardless of liver function (10–14 *vs.* <10 points: SHR 1.10; 95% CI 0.70–1.74; *p* = 0.690; ≥15 *vs.* <10 points: SHR 1.27; 95% CI 0.83–1.95; *p* = 0.270; Fig. 3C).

**Fig. 4:**
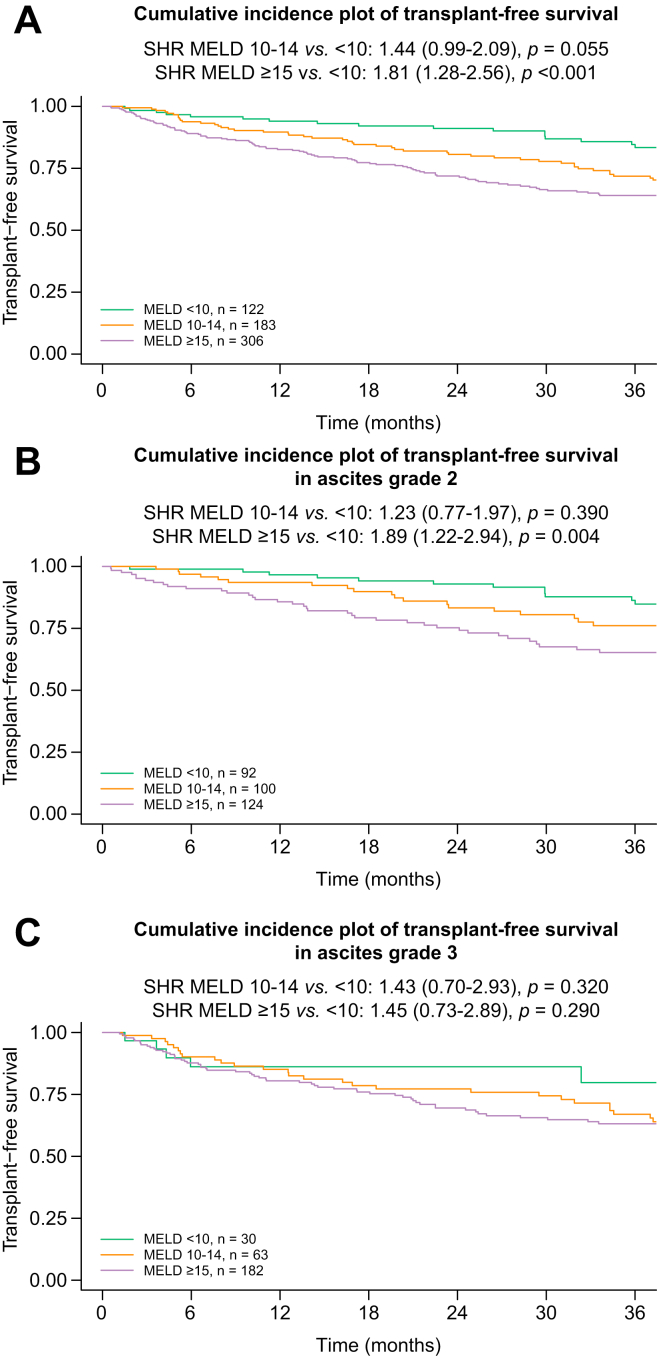
Transplant-free survival according to MELD and ascites severity at index presentation. Probability of transplant-free survival stratified according to MELD strata in (A) the study cohort, (B) patients with grade 2 ascites, and (C) patients with grade 3 ascites. MELD, model for end-stage liver disease; SHR, sub-distribution hazard ratio.

Overall, liver function was a determinant of TFS in competing risk analysis when stratifying patients according to MELD score (10–14 *vs.* <10 points: SHR 1.44; 95% CI 0.99–2.09; *p* = 0.055; ≥15 *vs.* <10 points: SHR 1.81; 95% CI 1.28–2.56; *p* <0.001; Fig. 4A). Patients with grade 2 ascites and a MELD score ≥15 points at index decompensation had an increased risk of transplant-free mortality, as compared with those with <10 points (10–14 *vs.* <10 points: SHR 1.23; 95% CI 0.77–1.97; *p* = 0.390; ≥15 *vs.* <10 points: SHR 1.89; 95% CI 1.22–2.94; *p* = 0.004; Fig. 4B). Moreover, in patients with ascites grade 3 at index decompensation, the incidence of transplant-free mortality was similarly high, regardless of MELD score (10–14 *vs.* <10 points: SHR 1.43; 95% CI 0.70–2.93; *p* = 0.320; ≥15 *vs.* <10 points: SHR 1.45; 95% CI 0.73–2.89; *p* = 0.290; Fig. 4C). Data were similar when stratified according to CPS, as provided in Figs. S5 and S6.

### Risk factors for mortality after ascites as the single index decompensation event

During a median follow-up of 48.5 (95% CI 40.0–57.2) months, 262 (42%) patients died with most deaths (n = 224, 85%) being considered liver-related. On univariable competing risk regression analysis with our 2 centres as a clustering variable, we could demonstrate that age and albumin were associated with mortality regardless of clustering between the 2 centres (Table 3). In multivariable analysis, age and albumin remained significantly associated with the outcome of interest. In multivariable analysis stratified to ascites grade, age, MELD score, and albumin were associated with mortality. Finally, age was associated with mortality in patients with grade 3 ascites in multivariable competing risk regression analysis (Table 3).

**Table 3:** **Risk factors for mortality in patients with ascites as the index decompensation event**.

Patient characteristics	Univariable		Overall cohort Model 1		Subgroup ascites grade 2 Model 2		Subgroup ascites grade 3 Model 3	
	SHR (95% CI)	*p* value	aSHR (95% CI)	*p* value	aSHR (95% CI)	*p* value	aSHR (95% CI)	*p* value
Age (year)	1.03 (1.01–1.05)	**<0.001**	1.04 (1.02–1.06)	**0.002**	1.04 (1.01–1.07)	**0.009**	1.04 (1.02–1.06)	**0.002**
Inclusion time								
2000–2013	1	–	–	–	–	–	–	–
2014–2021	1.17 (0.96–1.38)	0.140	–	–	–	–	–	–
MELD (point)	1.04 (0.99–1.09)	0.180	1.04 (0.97–1.11)	0.320	1.04 (1.02–1.06)	**0.002**	1.03 (0.93–1.13)	0.550
Albumin (g/L)	0.96 (0.94–0.98)	**<0.001**	0.98 (0.97–0.99)	**<0.001**	0.96 (0.94–0.98)	**<0.001**	0.99 (0.95–1.03)	0.600
Platelets (g/L)	1.00 (1.00–1.00)	0.830	–	–	–	–	–	–
Haemoglobin (g/L)	0.93 (0.79–1.07)	0.320	–	–	–	–	–	–

Univariable and multivariable competing risk regression analysis for mortality, including age, aetiological cure of liver disease, inclusion time, MELD, and albumin in the study cohort (model 1), grade 2 ascites (model 2), and grade 3 ascites (model 3). Values in bold denote statistical significance.

aSHR, adjusted subdistribution hazard ratio; MELD, model for end-stage liver disease; SHR, subdistribution hazard ratio.

## Discussion

Although ascites is the most frequent first decompensation event in patients with cirrhosis, there are hardly any studies on the natural history in this clinically relevant scenario. Thus, we used stringent inclusion criteria to systematically analyse the incidence and type of further decompensation in 622 patients with cirrhosis with ascites as the *single* first decompensation event.

The negative impact of ascites on liver-related outcomes in patients with cirrhosis is known,^1^ and some studies have reported on the clinical course of ascites.^1^^,^[19], [20], [21] However, our study is the first study that exclusively includes patients with *single* ascites decompensation (which easily explains why patients with concomitant decompensation events were excluded). Moreover, other studies describing the clinical course included fewer patients and other aetiologies of liver disease (*e.g.,* HBV or probable non-alcoholic steatohepatitis [NASH] cirrhosis focusing on the importance of a uniform and precise definition for the clinical diagnosis of NASH^20^) or also considered patients with ascites grade 1.^21^ Another study included only patients with ascites listed for LT and focused on short-term outcomes (*i.e.,* 90-day mortality^19^). All these studies did not focus on single ascites decompensation and are thus different to our study.

An hallmark study by D’Amico *et al.*,^1^ which focused on the index decompensating event, revealed that every 3rd patient with compensated cirrhosis experienced ascites as the primary decompensating event (33%), followed by variceal bleeding (10%) and death (10%). This study presented a prognostic sub-staging system for decompensated cirrhosis, whereas many previous studies simply discriminated patients with decompensated cirrhosis from those with compensated cirrhosis.^22^ However, the specific grade of ascites at first presentation was not further sub-classified. In our study, the overall incidence rate of variceal bleeding as further decompensation was low with only 54 events (9%) and with 27 (4%) bleeding events as the first further decompensation event. Importantly, further decompensation by variceal bleeding was not significantly different across Child–Pugh stages or ascites grades. The somewhat lower rate of variceal bleeding compared with that in the study by D’Amico *et al.*^1^ may be explained by a lower usage of non-selective beta-blocker use for primary bleeding prophylaxis.^23^ In 46% of patients with varices, non-selective beta-blocker therapy was already initiated at first ascites decompensation (n = 184/402). Interestingly, the rate of patients in primary bleeding prophylaxis with large varices was quite similar (n = 109/204, 53%)

As compared with variceal bleeding, non-bleeding further decompensation including HE, progression to refractory ascites, SBP, or HRS-AKI were far more frequent with 112 (18%), 130 (21%), 32 (5%), and 29 (5%) events, respectively. This finding suggests that although portal hypertension is involved in all these further decompensation events, the distinct risk of non-bleeding decompensation events is much higher if ascites is the *single* index decompensation event, whereas the risk for variceal bleeding may be lower in the absence of prior bleeding events.

Regarding a temporal sequence of further decompensation events, we tried to graphically depict the incidences of further decompensations using a Sankey plot (Fig. 2). There, we could show that the incidence of no further decompensation was 26%. Of course, patients who do not develop further decompensation/LT/death are more commonly presenting with grade 2 ascites. Incidences of further decompensating events are high, especially in patients with grade 3 ascites, namely, refractory ascites (21%) and HE (18%). Incidences of further decompensation remain equally distributed for the 2nd and 3rd decompensating events as in the first; however, incidences of death and LT are drastically increasing. We do think that there are patients with acutely decompensated liver disease who develop an ACLF triggered by infections/alcohol binge and have extremely high short-term mortality rates. As shown, ACLF is quite common in our study. However, there are also patients who do have a non-acute decompensation^24^ – also with grade 3 ascites – who develop multiple decompensating events, especially ascites-related. Eventually, patients may also have no further decompensation event after the 3rd, but those patients are only a few.

In general, acute decompensation and further decompensation are consequences of a complex pathomechanistic interplay between systemic inflammation and portal hypertension. Although differences in portal hypertension severity are most pronounced in patients with compensated cirrhosis, haemodynamic changes and cardiovascular (compensatory) pathomechanisms are distinct in decompensated stages and particularly in refractory ascites.^25^ Cardiac indices are highest in patients with ascites-related decompensation, indicating a pronounced hyperdynamic circulation with low systemic vascular resistance and splanchnic hyperperfusion potentially progressing to low mean arterial pressures.^25^ At some point, particularly when ascites becomes refractory, patients may show a reduction in cardiac output that is supposedly caused by insufficient cardiac inotropic and dromotropic compensation in advanced cirrhosis.^26^ In addition, systemic inflammation increases across clinical stages and is most pronounced in patients with decompensated cirrhosis.^27^ Thus, a systemic proinflammatory response combined with cardiocirculatory stress related to the profound portal hypertension-driven haemodynamic alterations may easily trigger further decompensation and ultimately increase the risk of death in cirrhosis.^25^^,^^27^

When comparing patients according to inclusion time, we found that patients who were included after 2014 were older and more commonly had alcohol-related liver disease and non-alcoholic fatty liver disease (NAFLD), whereas viral disease aetiologies were decreasing (Table S4). Although mean albumin levels were considerably lower, disease severity scores did not differ between the 2 time periods.

The differences between our 2 centres in disease aetiology are explainable when considering geographical differences in Italy and Austria (Table S5). Interestingly, patients from Padova were significantly healthier according to disease severity scores (*i.e.,* CPS 8 [IQR 7–9] *vs.* 9 [IQR 7–10]; *p* <0.001). Because we adjusted for potential clustering in our multivariable competing risk regression model,^18^ we are confident that our conclusions are firm.

In patients with grade 2 ascites, the rate of further decompensation is rather low with 18% and 29% after 1 and 3 years, respectively. In these patients, the degree of hepatic dysfunction, that is, MELD ≥15, still discriminates a subset of patients with a particularly high risk of further decompensation and mortality. Although this seems obvious at first sight, the consequences of this finding are extremely important in a clinical context: we suggest that in patients with grade 2 ascites, the risk stratification should always consider MELD, as patients with MELD ≥15 points show the highest risk for further decompensation and for mortality (Fig. S7). In contrast, in patients with grade 2 ascites with preserved liver function, particularly if MELD is <10, conservative management with diuretics and eventually paracentesis may represent the best management strategy as the risk for further decompensation and mortality seems low. In turn, patients with grade 2 ascites but MELD ≥15 may be evaluated early for LT candidacy and/or for TIPS. However, although incidences of further decompensation were not significantly different when comparing patients with grade 2 ascites and MELD <10 *vs.* 10–14, the incidences of LT/non-liver-related death (*i.e.*, the competing risks) tended to be higher in patients with MELD 10–14.

Somsouk *et al.*^19^ proposed that a MELD threshold at >21 points predicts short-term mortality. Because only some patients recruited for our study required/qualified for LT, the MELD threshold that was identified in our study to be prognostic for further decompensation was lower at MELD >15. This, again, underlines that we have included a different (*i.e.,* specifically earlier-staged) cohort of patients with ascites. Accordingly, we focused specifically on prediction of further hepatic decompensation/death during long-term follow-up, rather than on short-term follow-up.

Importantly, the 1-year incidence rates for further decompensation and mortality in patients presenting with grade 3 ascites at index decompensation are high (64% and 17%, respectively) across all MELD strata. The dismal clinical prognosis in patients with grade 3 ascites calls for intensified treatment strategies with disease-modifying drugs/interventions (*e.g.,* TIPS placement^28^ or investigational therapies such as long-term albumin administration^15^) as well as early access to LT.

TIPS placement has been demonstrated to improve the outcomes of patients with recurrent ascites^29^ but is commonly used (too) late, although data on improved control of ascites after (pre-emptive) TIPS implantation is promising.^16^^,^^28^ Future prospective studies should investigate earlier TIPS implantation in patients with grade 3 ascites at index decompensation and in patients with grade 2 ascites and high MELD (*i.e.,* ≥15 points).

Not surprisingly, age and albumin were associated with mortality even when adjusting for covariables and clustering. The same relies for the subgroup of ascites grade 2 where also the MELD score was associated with the outcome of interest, underlining the significant implications of the MELD score in risk prediction of patients with grade 2 ascites. Finally, only age was associated with mortality in patients with grade 3 ascites, independent of the MELD score.

Our Baveno cooperation-endorsed study describing the natural course of patients presenting with ascites as the *single* index decompensation has several strengths. First, it has a multicentre design that is based on data from a well-characterised inception cohort of patients with cirrhosis and ascites as the first decompensation event. Second, in contrast to most previous studies, we discriminated between grade 2 and grade 3 ascites at index decompensation. Our results demonstrate that graduation of ascites provides additional important information for clinical risk stratification, independently from liver function as indicated by MELD strata. Moreover, the impact of hepatic function (*i.e.,* especially albumin) on the clinical outcomes is distinct for different ascites severity grades. Third, the follow-up period with a median duration of 48.5 months (*i.e.,* more than 4 years) enabled a detailed evaluation of the different (competing) events that may occur after the first decompensation caused by ascites.

This study also has limitations. Owing to the large cohort of eligible patients (n = 3,377) and the relatively low included number of patients (n = 622), we cannot exclude selection bias. However, most patients were excluded because of compensated liver disease throughout the study period or other/multiple first decompensating events during follow-up (and thus, this cannot be regarded as selection bias). Furthermore, the exclusion of patients with prior LT and hepatic or extrahepatic malignancies seems justified, as their clinical outcomes depends on several important cofactors that are not present in the included patients with ascites only. Next, owing to the retrospective design of the study, we cannot exclude that some hepatic decompensation events have been missed. However, we have thoroughly reviewed all individual electronic health records of the hospital associations and nationwide electronic health records in both study centres. In addition, we have also performed searches of the LT database of our institutions and queried the nationwide death registries, which should largely avoid missing relevant clinical events.

In conclusion, our study systematically describes the natural history after ascites as a *single* decompensating event in patients with cirrhosis. Further decompensation most commonly occurs through progression to refractory ascites, SBP, HRS-AKI, and HE but only rarely because of variceal bleeding. Importantly, risk stratification, and thus the selection of most appropriate treatment strategies, must consider both the initial grade of ascites and MELD: patients with grade 2 ascites and low MELD (<15 points) showed a favourable prognosis and may be managed conservatively by diuretics and/or repetitive paracentesis. Patients with grade 3 ascites at index presentation showed considerably higher rates of further decompensation and mortality and thus may be considered early for intensified treatment such as TIPS or liver transplantation regardless of MELD.

## Financial support

No financial support specific to this study was received.

## Authors’ contributions

Concept of the study: LB, MTo, GS, SP, TR. Data collection: LB, MTo, GS, SP, TR. Statistical analysis: LB, GS. Drafting of the manuscript: LB, MTo, GS, SP, TR. Revision for important intellectual content and approval of the final manuscript: All authors.

## Data availability statement

The data that support the findings of this study are available from the corresponding author upon reasonable request.

## Conflicts of interest

The authors have nothing to disclose regarding the work under consideration for publication. The following authors disclose conflicts of interests outside the submitted work: LB, MTo, GS, VC, LH, SI, MJ, BSH, CGC, AA, AB, and SP have nothing to disclose. DB received travel support from AbbVie and Gilead and speaker fees from AbbVie and Siemens, as well as grant support form Gilead and Siemens. MTr received grant support from Albireo, Alnylam, Cymabay, Falk, Gilead, Intercept, MSD, Takeda, and UltraGenyx; honouraria for consulting from Albireo, Boehringer Ingelheim, BiomX, Falk, Genfit, Gilead, Intercept, Janssen, MSD, Novartis, Phenex, Pliant, Regulus, and Shire; speaker fees from Bristol-Myers Squibb, Falk, Gilead, Intercept, and MSD; and travel support from AbbVie, Falk, Gilead, and Intercept. MM served as a speaker and/or consultant and/or advisory board member for AbbVie, Collective Acumen, Gilead, and W.L. Gore & Associates and received travel support from AbbVie and Gilead. TR received grant support from AbbVie, Boehringer-Ingelheim, Gilead, Intercept, MSD, Myr Pharmaceuticals, Philips Healthcare, Pliant, Siemens, and W.L. Gore & Associates; speaking honoraria from AbbVie, Gilead, Gore, Intercept, Roche, and MSD; consulting/advisory board fees from AbbVie, Bayer, Boehringer-Ingelheim, Gilead, Intercept, MSD, and Siemens; and travel support from AbbVie, Boehringer-Ingelheim, Gilead, and Roche.

Please refer to the accompanying ICMJE disclosure forms for further details.
