# Supplementary material for: Risk of further decompensation/mortality in patients with cirrhosis and ascites as the first single decompensation event
Source: JHEP Rep. 2022 Jun 3;4(8):100513. doi: 10.1016/j.jhepr.2022.100513 (PMC9284386; doi:10.1016/j.jhepr.2022.100513)
Supplement: Multimedia component 1 [file mmc1.pdf]

Risk of further decompensation/mortality in patients with cirrhosis and ascites as the first single decompensation event

Lorenz Balcar, Marta Tonon, Georg Semmler, Valeria Calvino, Lukas Hartl, Simone Incicco, Mathias Jachs, David Bauer, Benedikt Silvester Hofer, Carmine Gabriele Gambino, Antonio Accetta, Alessandra Brocca, Michael Trauner, Mattias Mandorfer, Salvatore Piano, Thomas Reiberger, Baveno Cooperation: an EASL consortium

Table of contents

Table S1 2
Table S2 3
Table S3 4
Table S4 5
Table S5 6
Fig. S1 7
Fig. S2 8
Fig. S3 9
Fig. S4 10
Fig. S5 11
Fig. S6 12
Fig. S7 13

Table S1

	Grade-2 moderate ascites (n=323)	Grade-3 severe ascites (n=299)	SHR (95%CI) Grade 3 vs. 2	p-value
**Child-Pugh Score – median (IQR)**	7 (6-9)	9 (8-10)	-	-
**MELD – mean ± SD**	13.6±5.4	16.8±6.0	-	-
**Non-selective betablocker, n (%)**	119 (37%)	105 (31%)	-	-
**Diuretics, n (%)**	277 (86%)	277 (93%)	-	-
- **Furosemide**	29 (9%)	24 (8%)		
- **Antialdosteronic**	67 (21%)	58 (19%)		
- **Both**	181 (56%)	195 (65%)		
**Any further decompensation, n (%)**	121 (37%)	229 (77%)	3.64 (2.93-4.51)	**<0.001**
**First further decompensation event, n (%)**			-	-
- **Refractory ascites**	18 (6%)	112 (37%)		
- **SBP**	11 (3%)	21 (7%)		
- **HRS-AKI**	9 (3%)	20 (7%)		
- **Variceal bleeding**	18 (6%)	9 (3%)		
- **Hepatic encephalopathy**	55 (17%)	57 (19%)		
- **≥2 simultaneous further decompensation events**	10 (3%)	10 (3%)		
**All further decompensation events, n (%)**				
- **Refractory ascites**	39 (12%)	165 (55%)	6.90 (4.92-9.67)	**<0.001**
- **SBP**	30 (9%)	75 (25%)	3.36 (2.22-5.08)	**<0.001**
- **HRS-AKI**	24 (7%)	57 (19%)	2.96 (1.86-4.72)	**<0.001**
- **Variceal bleeding**	26 (8%)	28 (9%)	1.42 (0.82-2.45)	0.210
- **Hepatic encephalopathy**	73 (23%)	110 (37%)	2.08 (1.56-2.78)	**<0.001**
**Hyponatremia[1], n (%)**	86 (27%)	129 (43%)	2.08 (1.59-2.71)	**<0.001**
**ACLF, n (%)**	46 (14%)	100 (33%)	3.08 (2.18-4.36)	**<0.001**
**PVT, n (%)**	34 (11%)	28 (9%)	1.01 (0.62-1.65)	**<0.001**
**HCC, n (%)**	53 (16%)	26 (9%)	0.63 (0.39-1.00)	**0.048**
**TIPS, n (%)**	28 (9%)	53 (18%)	-	-
**LT, n (%)**	52 (16%)	53 (18%)	-	-
**Death, n (%)**	126 (39%)	136 (45%)	1.40 (1.10-1.79)	**0.006**
- **Liver-related**	102 (81%)	122 (90%)	-	-
- **Non-liver-related**	22 (17%)	14 (10%)		
- **Unknown**	2 (2%)	-		

[1] defined as serum sodium <130 mmol x L^{-1}

Table S1. Follow-up characteristics compared between patients with grade-2 and grade-3 ascites.

Abbreviations: n number; ACLF acute-on-chronic liver failure; HCC hepatocellular carcinoma; HRS-AKI hepatorenal syndrome-acute kidney injury; IQR interquartile range; OLT orthotopic liver transplantation; PVT portal vein thrombosis; SBP spontaneous bacterial peritonitis; SHR subdistribution hazard ratio; TIPS transjugular intrahepatic portosystemic shunt

Table S2

Variable	CPS-A (n=93)	CPS-B (n=359)	CPS-C (n=170)	p-value
**Any further decompensation, n (%)**	24 (26%)	207 (58%)	119 (70%)	**<0.001**
**First further decompensation event, n (%)**				
- **Refractory ascites**				
- **SBP**	4 (4%)	81 (23%)	45 (26%)	
- **HRS-AKI**	1 (1%)	25 (7%)	6 (4%)	**<0.001**
- **Variceal bleeding**	1 (1%)	11 (3%)	17 (10%)	
- **Hepatic encephalopathy**	3 (3%)	19 (5%)	5 (3%)	
- **More than one**	13 (14%)	59 (16%)	40 (24%)	
**decompensation event**	2 (2%)	12 (3%)	6 (4%)	
**All further decompensation events (occuring ever), n (%)**				
- **Refractory ascites**	10 (11%)	122 (34%)	72 (42%)	**<0.001**
- **SBP**	4 (4%)	68 (19%)	33 (19%)	**0.002**
- **HRS-AKI**	4 (4%)	42 (12%)	35 (21%)	**<0.001**
- **Variceal bleeding**	4 (4%)	35 (10%)	15 (9%)	0.250
- **Hepatic encephalopathy**	16 (17%)	97 (27%)	70 (41%)	**<0.001**
**Hyponatremia[1], n (%)**	17 (18%)	116 (32%)	82 (48%)	**<0.001**
**Number of paracenteses, median (IQR)**	0 (0-1)	1 (0-5)	2 (0-5)	**<0.001**
**ACLF, n (%)**	7 (8%)	77 (21%)	62 (36%)	**<0.001**
**PVT, n (%)**	12 (13%)	36 (10%)	14 (8%)	0.481
**HCC, n (%)**	19 (20%)	48 (13%)	12 (7%)	**0.007**
**TIPS, n (%)**	9 (10%)	54 (15%)	18 (11%)	0.212
**OLT, n (%)**	14 (15%)	60 (17%)	31 (18%)	0.798
**Death, n (%)**	28 (30%)	162 (45%)	72 (42%)	**0.033**
**Type of deaths, n (%)**				
- **Liver-related**	22 (79%)	136 (84%)	66 (92%)	
- **Non-liver related**	5 (18%)	25 (15%)	6 (8%)	**<0.001**
- **Unknown**	1 (4%)	1 (1%)	-	

[1] defined as serum sodium <130 mmol x L^{-1}

Table S2. Follow-up characteristics compared among CPS stages.

Abbreviations: n number; ACLF acute-on-chronic liver failure; HCC hepatocellular carcinoma; HRS-AKI hepatorenal syndrome-acute kidney injury; IQR interquartile range; OLT orthotopic liver transplantation; PVT portal vein thrombosis; SBP spontaneous bacterial peritonitis; TIPS transjugular intrahepatic portosystemic shunt

Table S3

	MELD <10 (n=126)	MELD 10-14 (n=237)	MELD ≥15 (n=259)	p-value
**Any further decompensation, n (%)**	49 (39%)	126 (53%)	175 (68%)	**<0.001**
**First further decompensation event, n (%)**				**<0.001**
- **Refractory ascites**	14 (11%)	49 (21%)	67 (26%)	
- **SBP**	8 (6%)	16 (7%)	8 (3%)	
- **HRS-AKI**	2 (2%)	4 (2%)	23 (9%)	
- **Variceal bleeding**	6 (5%)	9 (4%)	12 (5%)	
- **Hepatic encephalopathy**	14 (11%)	42 (18%)	56 (22%)	
- **More than one decompensation event**	5 (4%)	6 (3%)	9 (3%)	
**All further decompensation events, n (%)**				
- **Refractory ascites**	24 (19%)	72 (30%)	108 (42%)	**<0.001**
- **SBP**	15 (12%)	38 (16%)	52 (20%)	0.121
- **HRS-AKI**	10 (8%)	19 (8%)	52 (20%)	**<0.001**
- **Variceal bleeding**	7 (6%)	16 (7%)	31 (12%)	**0.045**
- **Hepatic encephalopathy**	22 (18%)	61 (26%)	100 (39%)	**<0.001**
**Hyponatremia[1], n (%)**	24 (19%)	73 (31%)	118 (46%)	**<0.001**
**Number of paracenteses, median (IQR)**	1 (0-3)	1 (0-5)	2 (0-6)	**0.002**
**ACLF, n (%)**	16 (13%)	41 (17%)	89 (34%)	**<0.001**
**PVT, n (%)**	15 (12%)	24 (10%)	23 (9%)	0.729
**HCC, n (%)**	17 (%)	33 (14%)	29 (11%)	0.632
**TIPS, n (%)**	12 (10%)	35 (15%)	34 (13%)	0.368
**OLT, n (%)**	14 (11%)	41 (17%)	50 (19%)	0.128
**Death, n (%)**	38 (30%)	103 (44%)	121 (47%)	**0.007**
**Type of deaths, n (%)**				0.257
- **Liver-related**	30 (79%)	87 (84%)	107 (88%)	
- **Non-liver related**	8 (21%)	14 (14%)	14 (12%)	
- **Unknown**	-	2 (2%)	-	

[1] defined as serum sodium <130 mmol x L^{-1}

Table S3. Follow-up characteristics compared among MELD strata.

Abbreviations: n number; ACLF acute-on-chronic liver failure; HCC hepatocellular carcinoma; HRS-AKI hepatorenal syndrome-acute kidney injury; IQR interquartile range; OLT orthotopic liver transplantation; PVT portal vein thrombosis; SBP spontaneous bacterial peritonitis; TIPS transjugular intrahepatic portosystemic shunt

Table S4

Patients – n (%)	Included < 2014 n=320 (51%)	Included ≥ 2014 n=302 (49%)	p-value
**Age (years) – mean ± SD**	55.0±11.2	58.1±11.0	**<0.001**
**Sex (M) – n (%)**	216 (68%)	207 (69%)	0.780
**Etiology[1]**			
**Alcohol – n (%)**	173 (54%)	193 (64%)	**0.013**
**HCV – n (%)**	92 (29%)	50 (17%)	**<0.001**
**HBV – n (%)**	42 (13%)	16 (5%)	**<0.001**
**NAFLD – n (%)**	28 (9%)	47 (16%)	**0.009**
**Autoimmune – n (%)**	15 (5%)	15 (5%)	0.871
**Other – n (%)**	13 (4%)	23 (8%)	0.058
**Ascites grade**			
**Grade-2/moderate – n (%)**	165 (52%)	158 (52%)	0.851
**Grade-3/severe – n (%)**	155 (48%)	144 (48%)	
**Varices[2] (yes vs. no) – n (%)**			
**None – n (%)**	98 (31%)	95 (32%)	
**Small – n (%)**	108 (34%)	90 (30%)	0.051
**Large – n (%)**	95 (30%)	109 (36%)	
**Child-Pugh Stage**			
**A – n (%)**	52 (16%)	41 (14%)	
**B – n (%)**	187 (58%)	172 (57%)	0.410
**C – n (%)**	81 (25%)	89 (30%)	
**Child-Pugh Score – median (IQR)**	8 (7-10)	8 (7-10)	0.455
**MELD – mean ± SD**	15.3±6.0	14.9±5.7	0.407
**Non-selective betablocker, n (%)**	118 (37%)	106 (35%)	0.645
**Diuretics, n (%)**	274 (86%)	280 (93%)	
- **Furosemide**	22 (7%)	31 (10%)	0.017
- **Aldosterone antagonists**	67 (21%)	58 (19%)	
- **Both**	185 (58%)	191 (63%)	
**Albumin (g x L^{-1}) – mean ± SD**	32.7±6.2	31.5±5.9	**0.014**
**Bilirubin (mg x dL^{-1}) – median (IQR)**	2.01 (1.17-3.58)	1.92 (1.02-3.50)	0.367
**INR – mean ± SD**	1.41±0.32	1.42±0.30	0.544
**Platelets (G x L^{-1}) – median (IQR)**	103 (65-152)	122 (85-175)	**<0.001**

[1] patients may have more than one liver disease etiology
[2] missing information in 27 (4%) patients

Table S4. Comparison of patient characteristics according to time of inclusion (prior vs. after 2014).

Abbreviations: M male; F female; n number; Alcohol alcohol-related liver disease; HCV hepatitis C virus; HBV hepatitis B virus; NASH non-alcoholic steatohepatitis; SD standard deviation; MELD Model for end-stage liver disease; IQR interquartile range; INR international normalized ratio

Table S5

Patients – n (%)	Padova n=315 (51%)	Vienna n=307 (49%)	p-value
**Age (years) – mean ± SD**	56.9±11.9	56.1±10.5	0.342
**Sex (M) – n (%)**	215 (68%)	208 (68%)	0.893
**Etiology[1]**			
**Alcohol – n (%)**	162 (51%)	204 (66%)	**<0.001**
**HCV – n (%)**	90 (29%)	52 (17%)	**<0.001**
**HBV – n (%)**	48 (15%)	10 (3%)	**<0.001**
**NAFLD – n (%)**	44 (14%)	31 (10%)	0.138
**Autoimmune – n (%)**	19 (6%)	11 (4%)	0.154
**Other – n (%)**	10 (3%)	26 (9%)	**0.005**
**Ascites grade**			
**Grade-2/moderate – n (%)**	187 (59%)	136 (44%)	**<0.001**
**Grade-3/severe – n (%)**	128 (41%)	171 (56%)	
**Varices[2] (yes vs. no) – n (%)**			
**None – n (%)**	112 (36%)	81 (26%)	**<0.001**
**Small – n (%)**	118 (38%)	80 (26%)	
**Large – n (%)**	73 (24%)	131 (43%)	
**Child-Pugh Stage**			
**A – n (%)**	68 (22%)	25 (8%)	**<0.001**
**B – n (%)**	184 (58%)	175 (57%)	
**C – n (%)**	63 (20%)	107 (35%)	
**Child-Pugh Score – median (IQR)**	8 (7-9)	9 (7-10)	**<0.001**
**MELD – mean ± SD**	14.1±6.0	16.2±5.6	**<0.001**
**Non-selective betablocker, n (%)**	112 (36%)	106 (35%)	0.810
**Diuretics, n (%)**	254 (81%)	280 (93%)	**<0.001**
- **Furosemide**	27 (9%)	31 (10%)	
- **Aldosterone antagonists**	29 (9%)	58 (19%)	
- **Both**	198 (63%)	191 (63%)	
**Albumin (g x L^{-1}) – mean ± SD**	33.8±5.9	30.6±5.9	**<0.001**
**Bilirubin (mg x dL^{-1}) – median (IQR)**	1.57 (0.91-2.90)	2.30 (1.41-4.17)	**<0.001**
**INR – mean ± SD**	1.37±0.33	1.46±0.29	**<0.001**
**Platelets (G x L^{-1}) – median (IQR)**	104 (65-146)	126 (81-183)	**<0.001**

[1] patients may have more than one liver disease etiology
[2] missing information in 27 (4%) patients

Table S5. Baseline characteristics in the subgroups of patients from Padova (n=315) and Vienna (n=307).

Abbreviations: M male; F female; n number; HCV hepatitis C virus; HBV hepatitis B virus; NASH non-alcoholic steatohepatitis; SD standard deviation; CTP Child-Turcotte-Pugh score; MELD Model for end-stage liver disease; IQR interquartile range; INR international normalized ratio; PLT platelet count; SBP spontaneous bacterial peritonitis

Fig. S1

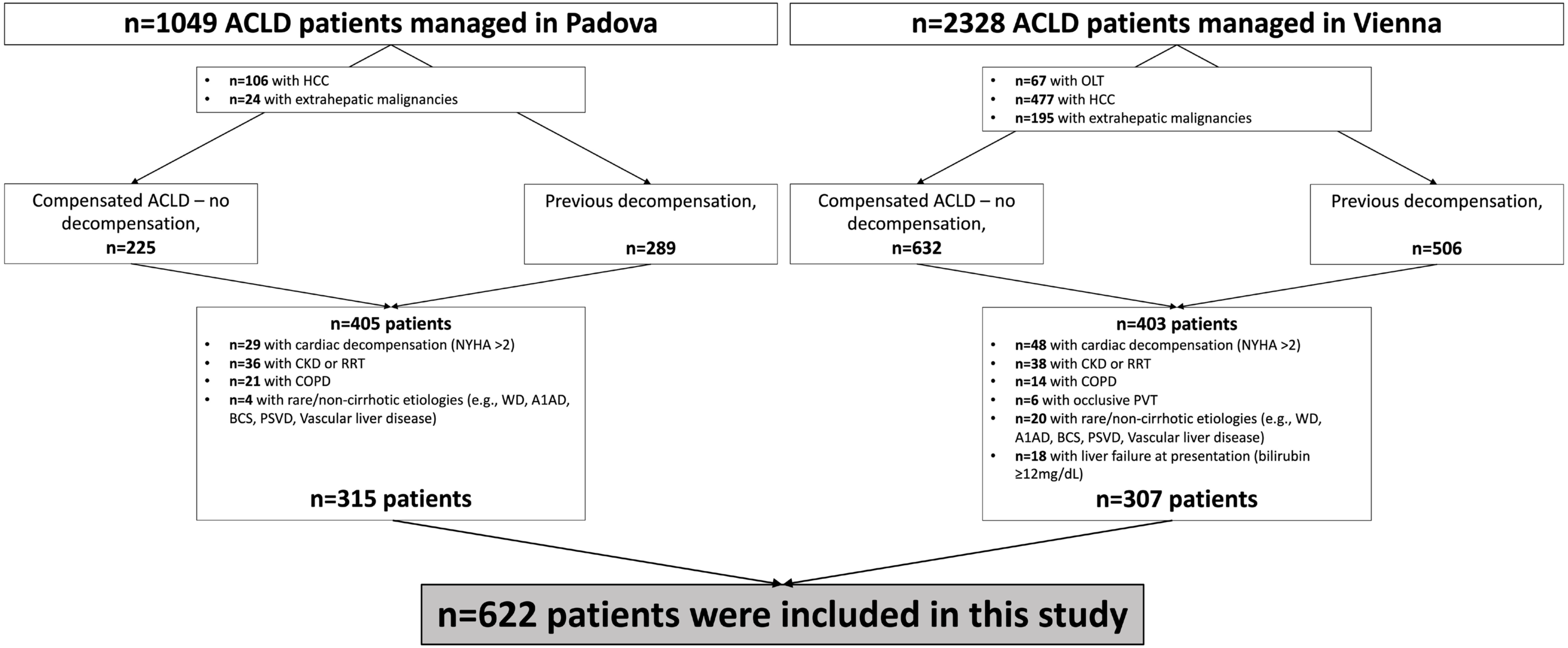

Fig. S1. Patient flowchart of the centres in Padova and Vienna.

Abbreviations: A1AD alpha-1 antitrypsin deficiency; ACLD advanced chronic liver disease; ACLF acute-on-chronic liver failure; BCS Budd-Chiari syndrome; BL baseline; CKD chronic kidney disease; COPD chronic obstructive pulmonary disease; HCC hepatocellular carcinoma; NYHA New York Heart Association; OLT orthotopic liver transplantation; PSVD porto-sinusoidal vascular disease; PVT portal vein thrombosis; RRT renal replacement therapy; WD Wilson's disease

Fig. S2

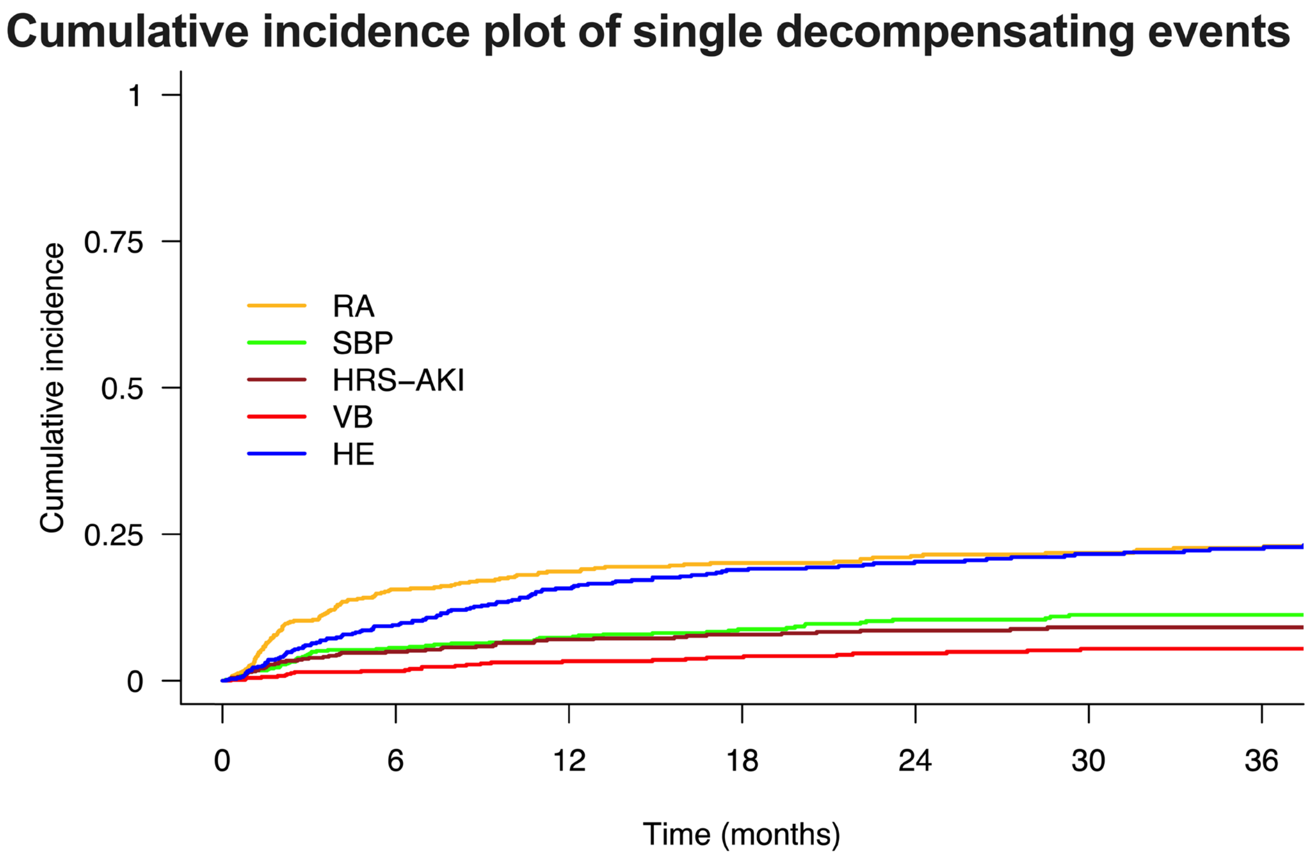

Fig. S2. Cumulative incidence plots of each first/further decompensation event.

Abbreviations: RA – refractory ascites; SBP – spontaneous bacterial peritonitis; HRS-AKI – hepatorenal syndrome-acute kidney injury; VB – variceal bleeding; HE – hepatic encephalopathy

Fig. S3

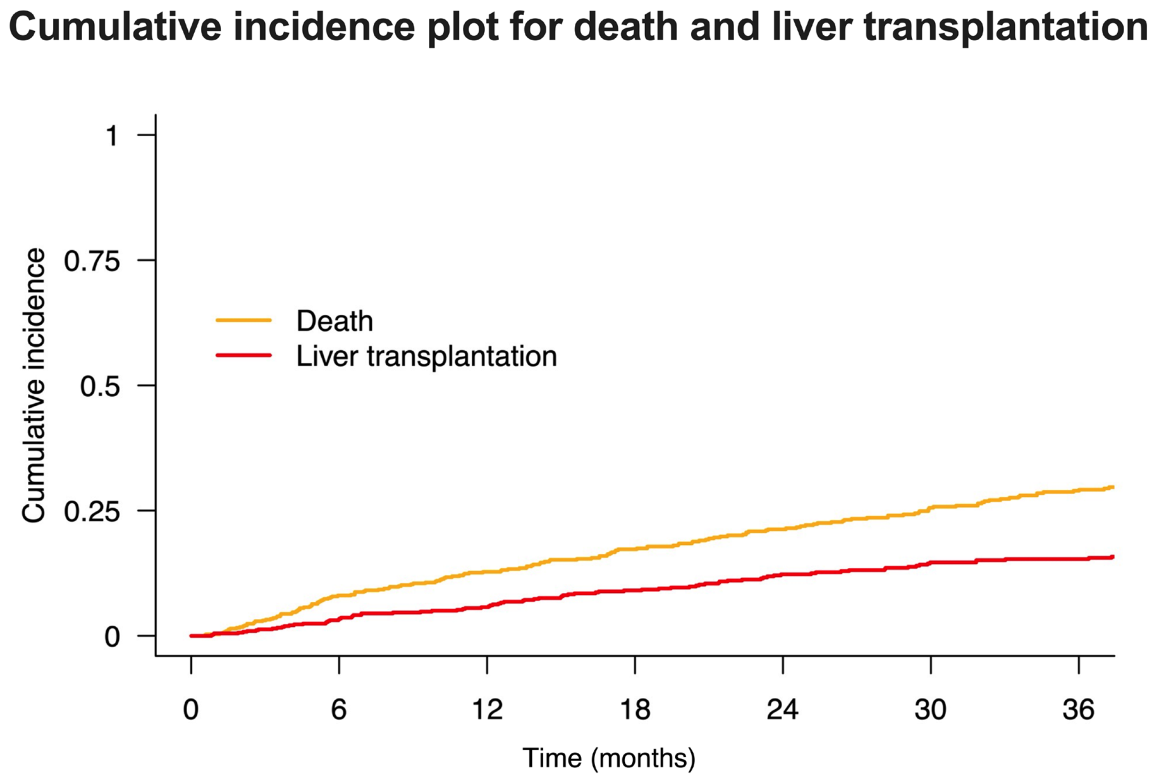

Fig. S3. Cumulative incidence plot for death and liver transplantation.

Fig. S4

[A] Cumulative incidence plot of further decompensation according to ascites grade

SHR: 3.64 (2.93-4.51), p<0.001

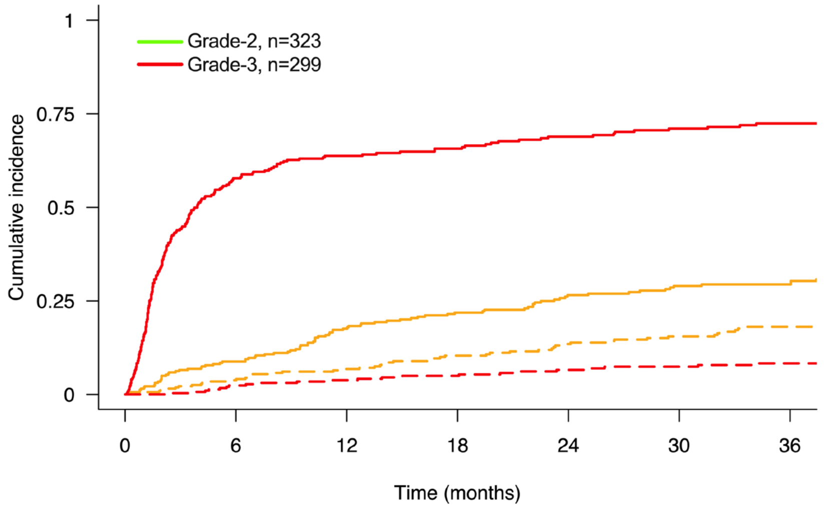

[B] Cumulative incidence plot of transplant-free survival according to ascites grade

SHR: 1.40 (1.10-1.79), p=0.006

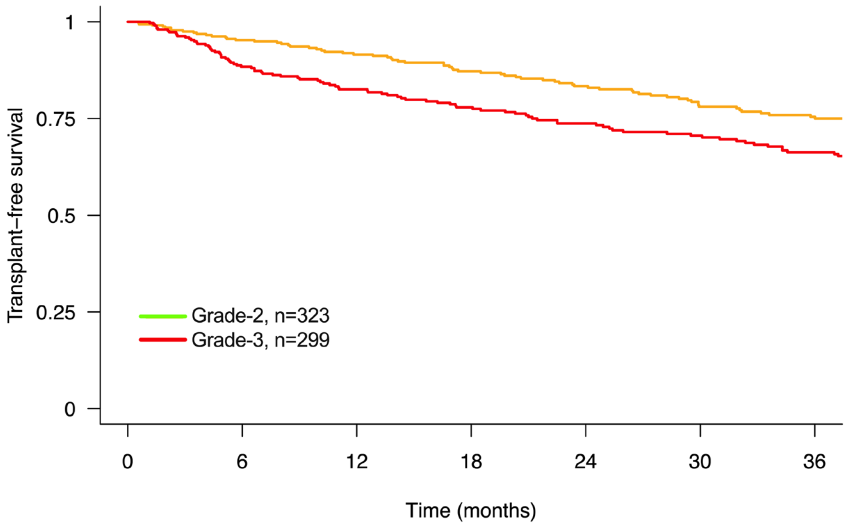

Fig. S4. Cumulative incidence plot of further decompensation (**A**) and probability of transplant-free survival (**B**) stratified according to the severity of ascites.

Fig. S5

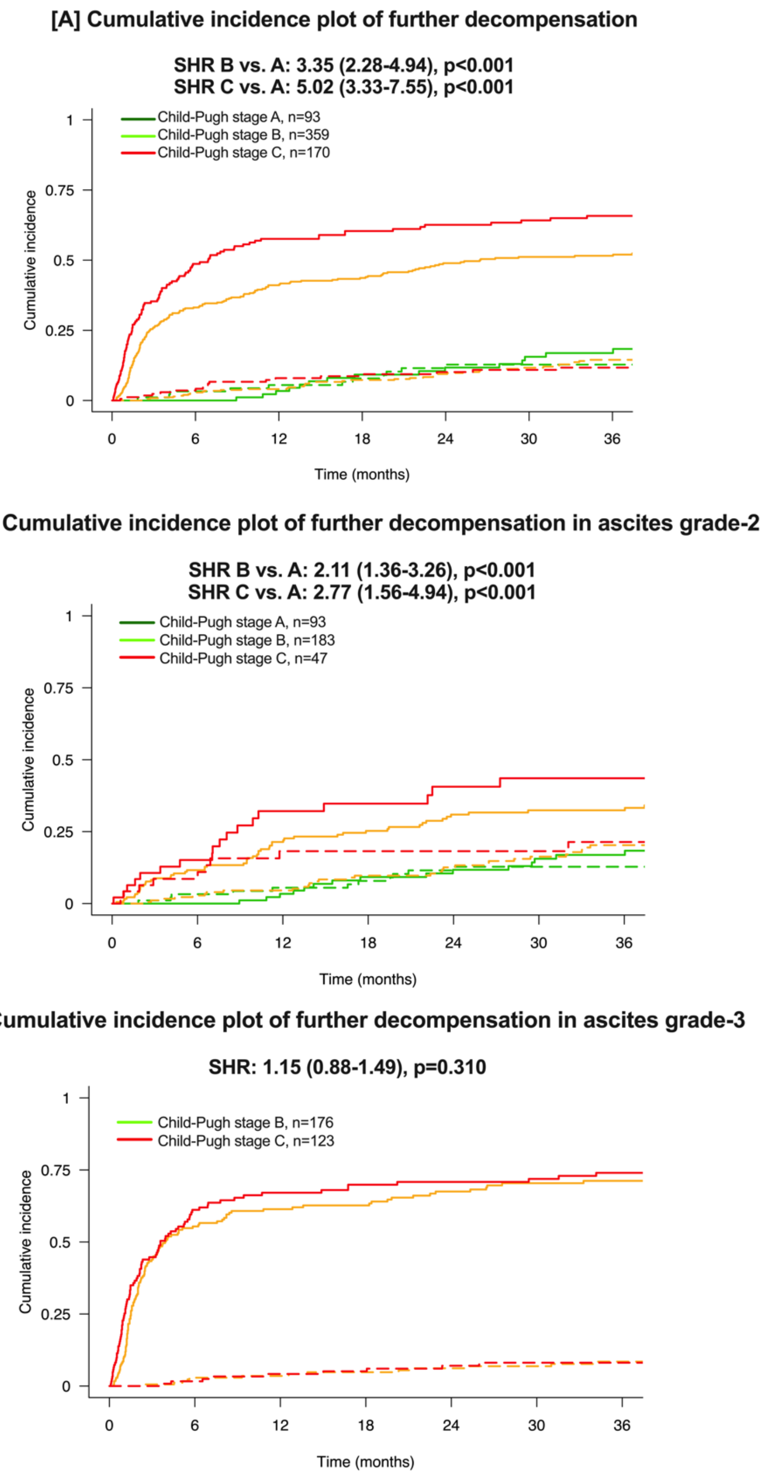

Fig. S5. Cumulative incidence plot of further decompensation stratified according to CPS stages in the study cohort (**A**), in patients with grade-2 ascites (**B**), and in patients with grade-3 ascites (**C**).

Fig. S6

[A] Cumulative incidence plot of transplant-free survival

SHR B vs. A: 1.81 (1.24-2.66), p=0.002
SHR C vs. A: 1.94 (1.26-2.97), p=0.003

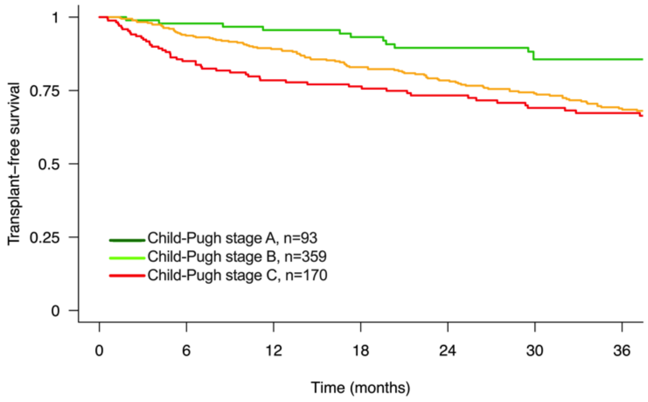

[B] Cumulative incidence plot of transplant-free survival in ascites grade-2

SHR B vs. A: 1.80 (1.19-2.73), p=0.006
SHR C vs. A: 1.32 (0.69-2.52), p=0.400

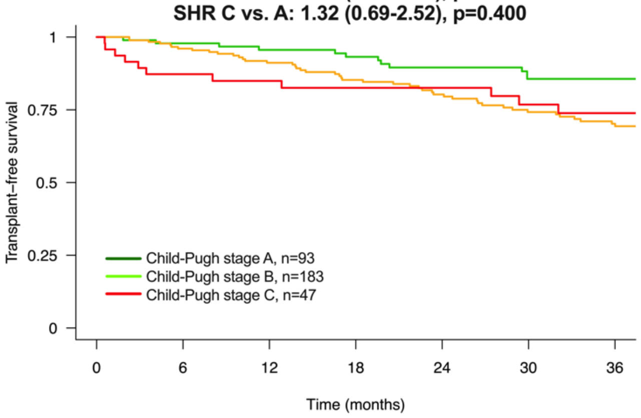

[C] Cumulative incidence plot of transplant-free survival in ascites grade-3

SHR: 1.17 (0.83-1.65), p=0.360

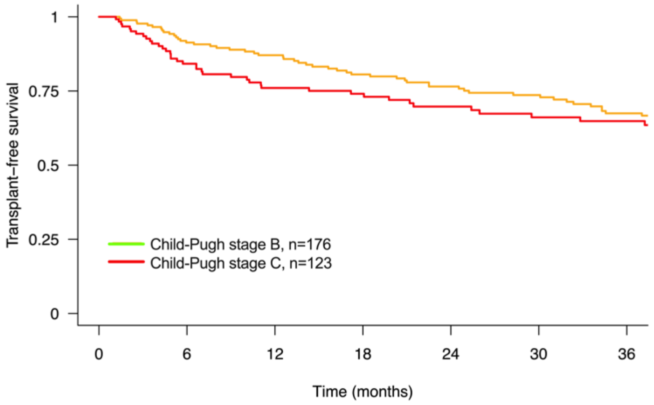

Fig. S6. Probability of transplant-free survival stratified according to CPS stages in the study cohort (**A**), in patients with grade-2 ascites (**B**), and in patients with grade-3 ascites (**C**).

Fig. S7

[A] Cumulative incidence plot of further decompensation in patients with ascites grade-2

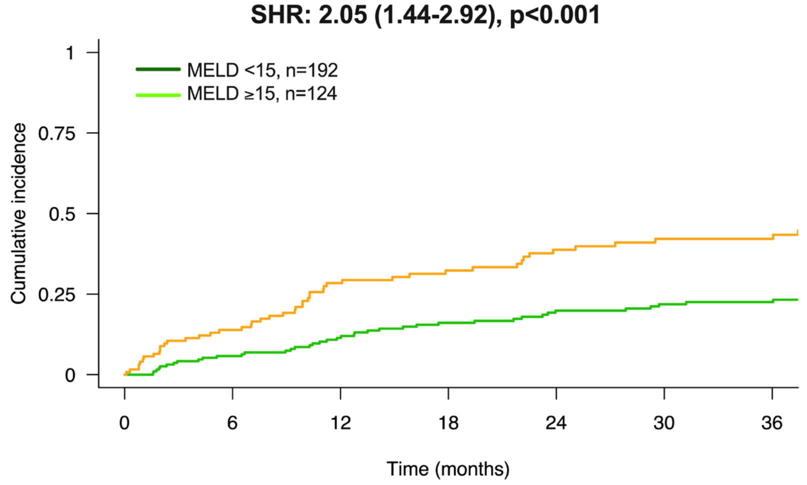

[B] Cumulative incidence plot of transplant-free survival in patients with ascites grade-2

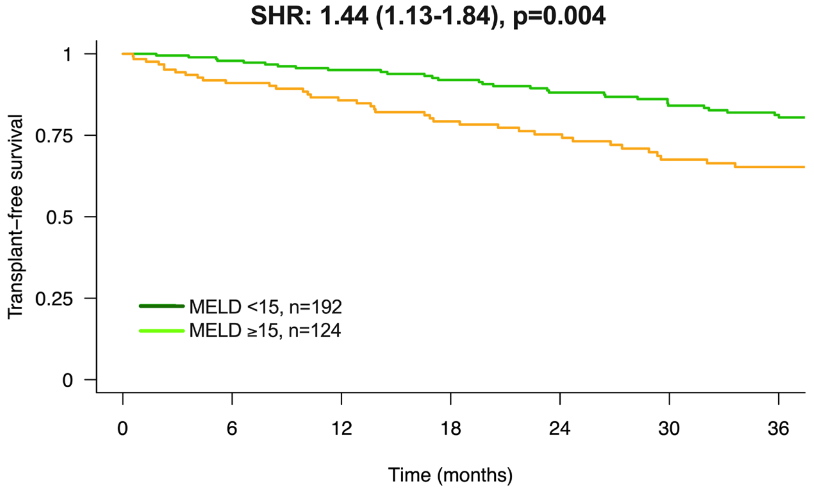

Fig. S7. Cumulative incidence plot of further decompensation (**A**) and transplant-free survival (**B**) in patients with grade-2 ascites stratified by MELD <15 vs. ≥15.
